# Socio-Demographic Factors, Gambling Behaviour, and the Level of Gambling Expenditure: A Population-Based Study

**DOI:** 10.1007/s10899-021-10075-6

**Published:** 2021-10-04

**Authors:** Tanja Grönroos, Anne Kouvonen, Jukka Kontto, Anne H. Salonen

**Affiliations:** 1 Department of Public Health and Welfare, Finnish Institute for Health and Welfare, Helsinki, Finland; 2 Faculty of Social Sciences, University of Helsinki, Helsinki, Finland; 3 Centre for Public Health, Queen’s University Belfast, Belfast, UK; 4 Faculty of Health Sciences, University of Eastern Finland, Kuopio, Finland

**Keywords:** Gambling expenditure, Gambling, Longitudinal study, Population survey, Register data, Socio-demographic factors

## Abstract

The aim of this study was to examine the relationship between socio-demographic factors, gambling behaviour, and the level of gambling expenditure. The data were drawn from the population-based Gambling Harms Survey 2016 and 2017 conducted in Finland. The data were linked to register-based variables. Past-year gamblers were included (Wave 1; n = 5 805, both Waves; n = 2 165). The study showed that of the 4.2 % of gamblers that produced 50.0 % of the total GE in 2016, 33.1 % of the GE was produced by those with a gambling problem and 43.3 % by those with at-risk gambling pattern. Compared to gamblers in the lowest GE group, those in the highest GE group were more likely to be men, aged 25 or older, with upper secondary education, have a high income, be on disability pension or sickness allowance, be frequent gamblers, gambling at least six game types, and showing at-risk and problem gambling patterns. Cumulative weekly GE by income tertiles remained fairly stable between the years. The results suggest that GE is highly concentrated. Among the small group of high-intensity consumers, the majority of the revenue comes from at-risk and problem gambling. Participants in the low GE group differ from those in the intermediate and high GE groups in terms of socio-demographics and gambling behaviour.

**Supplementary Information:**

The online version contains supplementary material available at 10.1007/s10899-021-10075-6.

## Introduction

Gambling environment has changed rapidly. Gambling is increasingly available and more advertised, and new gambling products are constantly being developed. People also spend more money on gambling than before (Statista 2020). Finland’s per capita gambling expenditure (GE) rate is one of the highest in Europe (Economist 2017). Spending for gambling is highly concentrated (Salonen et al., 2017; Salonen et al., 2019; Salonen et al., 2020; Williams et al., 2011). This raises a question of how much of the gambling revenue is produced by individuals from different socio-demographic backgrounds and, especially, by those with a gambling problem.

Overall, men tend to spend more money on gambling than women (McCormack et al., 2014; Davidson et al., 2016; Castrén et al., 2018). This has been observed for both younger and older adults (Merkouris et al., 2016; Molinaro et al., 2018). GE increases with age (Tan et al., 1991), although some studies suggest that GE increases only until the middle age and starts to drop after that (Mikesell 1991; Scott & Garen 1994).

Moreover, low socio-economic status is associated with high GE (Davidson et al., 2016; Salonen et al., 2018a). To date, a limited number of studies have investigated the relationship between GE and receipt of social security benefits (Worthington 2001; MacDonald et al., 2004). A Canadian survey showed that households with income support were less likely to gamble. With the exception of one jurisdiction, households that received income support spend a lower proportion of their income on gambling. (MacDonald et al., 2004.) However, gambling problems are more common among those who receive income support or who are unemployed (McMillen et al., 2004). Moreover, gambling opportunities are concentrated in the socio-economically disadvantaged areas (Wardle et al., 2014; Raisamo et al., 2019).

Studies conducted in different countries have shown that although high income groups spend more on gambling, lower income groups contribute proportionally more (Beckert & Lutter 2009; Canale et al., 2016; Castrén et al., 2018; Roukka & Salonen 2020). In general, among those with low income, a large fraction of their income spent on gambling often leads to financial harm (Welte et al., 2004; Freund & Morris 2006), since they have fewer financial resources to cover up their losses. This may lead to debt taking (Sulkunen et al., 2019). A Finnish population survey showed that among help-seeking gamblers, 42 % had a debt problem or a debt spiral (Salonen et al., 2018b). Gambling-related harms (GRH) include financial consequences, such as over-indebtedness or losing funds intended for household expenses. In some cases, gamblers may lose all their assets, even their homes (Sulkunen et al., 2019). In addition, gambling may cause work-related problems and job loss; housing instability or homelessness; and criminal behaviour (Williams et al., 2011; Gattis & Cunninham-Williams 2011; Lind et al., 2015; Eby et al., 2016).

High gambling frequency is associated with high GE (LaPlante et al., 2009). The number of game types gambled affects GE, as those who gamble six or more game types spend the most. Furthermore, online gambling is associated with higher GE than land-based gambling (Salonen et al., 2017). Weekly gambling on horse races, online poker, EGMs and offshore games are linked with higher GE (Salonen et al., 2018b). EGMs cause more GRHs than other game types (Peluuri 2016; Binde et al., 2017).

High GE does not directly mean that a gambler experience GRH, but it is associated with GRH (Currie et al., 2006; Langham et al., 2016). A relatively large proportion of the gambling revenue comes from those with a gambling problem (e.g., Grinols & Omorov 1996; Lesieur 1998; Volberg et al., 1998; Volberg et al., 2011; Williams & Wood 2004; Wood & Williams 2007; Salonen et al., 2017; Fiedler et al., 2019). However, in previous studies, this proportion have ranged widely from 14 % (Volberg et al., 2011) to 52 % (Grinols & Omorov 1996). In addition to several methodological differences, the proportion of gambling revenue derived from those with a gambling problem depends on the game type, with lower proportions for lotteries and higher proportions for electronic gaming machines (EGM) (Volberg et al., 1998; Wood & Williams 2007).

Until 2016, the Finnish gambling policy was based on a three-party monopoly system. These operators were merged into a single state-owned company in 2017. Under the Lotteries Act, the country’s gambling system’s main objective is to prevent and reduce the gambling-related financial, health and social harm (Finlex 2011). Most of the Finnish gambling company’s profits are channelled through the state or non-governmental organisations to promote the public good. The use of gambling revenues for good purposes is often used as a justification for the existence of this monopoly, in which all citizens are seen as beneficiaries.

As discussed above, previous studies have investigated the association between gambling expenditure, socio-demographic factors, and gambling behaviour. Nevertheless, very little is known about the small group of gamblers that produces the most of the gambling revenue. This study aims to investigate the relationship between socio-demographic factors (education, employment status, net income, and social security benefits), gambling behaviour (gambling frequency, number of game types gambled, gambling mode, and gambling severity), and the level of GE. The gamblers with the highest GE are compared to other gamblers.

## Methods

### Design and Participants

The data were drawn from the Gambling Harms Survey 2016–2017 conducted in Finland (Salonen et al., 2019). Finland is the only European Union country that still maintains a gambling monopoly; this makes Finland an interesting case. In addition, these data are longitudinal, which allows us to analyse individual-level changes. At Wave 1, a total of 20,000 permanent residents of selected areas were randomly selected from the national Population Information System. The inclusion criteria were: (1) age 18 or over; (2) Finnish or Swedish as first language; and (3) living in Uusimaa, Pirkanmaa or Kymenlaakso. At Wave 1, a total of 4 611 participants gave their permission to be re-contacted.

### Data Collection

The study was conducted by the Finnish Institute for Health and Welfare in collaboration with the University of Helsinki. The data were collected by Statistics Finland between 9 January and 26 March 2017 (Wave 1) and between 15 January and 30 April 2018 (Wave 2) by using online and postal survey. The focus was on gambling in 2016 and 2017.

At Wave 1, a participant information sheet was sent to the potential participants. The first reminder was sent by mail. To 25–44-years-olds, the reminder was also sent by text message. The third and fourth reminder included a postal survey. At Wave 2, the postal survey was sent to participants that had responded via postal survey at Wave 1. Similarly, an invitation with two reminders was sent to those participants that had answered to online survey at Wave 1. All invitations included a link to online survey.

At Wave 1, a total of 7 186 persons participated in the survey (response rate 36 %). At Wave 2, there were 2 624 participants, and the response rate was 57 % of eligible participants, who responded to Wave 1 survey and gave a permission for re-contact. At Wave 1, the response rate was lower in men than in women (Salonen et al., 2017); whereas at Wave 2 men responded more actively than women (Salonen et al., 2019). Overall, older and highly educated persons participated most actively.

Past-year gamblers were included (Wave 1; n = 5 805, both Waves; n = 2 165). Using national ID numbers, the survey data were linked to the Statistics Finland’s register data on socio-demographic factors.

### Measures

#### Gambling Expenditure

GE was measured as follows: ‘Think about the year 2016 (or 2017). Estimate the amount of money that you spent on gambling on average per week, per month or during the year (in Euros).’ All GE figures were transformed into weekly GE. To categorise three groups, the cumulative distribution of total GE was created by sorting GE in descending order. Then, the cumulative distribution was explored to find the point where half of the total GE was cumulated. Lastly, based on the Pareto Principle (Juran 1975), which assumes that for many phenomena roughly 80 % of consequences are due to 20 % of the causes, the cumulative distribution was explored to find the point where 80 % of total GE was cumulated. Gamblers were divided into three groups as follows: (1) produces 50 % of the total GE and spend EUR ≥ 40.0 per week (i.e., the highest GE group), (2) produces 30 % of the total GE and spend EUR 10.0–39.9 per week (i.e., the intermediate GE group), and (3) produces 20 % of the total GE and spend EUR ≤ 9.9 per week (i.e., the lowest GE group).

The highest GE at Wave 2 was considered as an outlier and replaced as follows: firstly, all gamblers in both Waves with increased GE were selected from the dataset. Then, the average growth of GE between the waves was calculated for them. The outlier was replaced by multiplying the respondents’ GE in Wave 1 by the average growth of GE.

#### Socio-Demographic Factors

Socio-demographic factors were derived from Statistics Finland’s registers and recoded (Table 1). They included gender, age, educational level, employment status, and personal net income tertiles. Furthermore, the amount of income support, unemployment benefit and sickness allowance were dichotomised (yes/no). In Finland, income support is a last-resort form of financial aid, which covers some of the basic necessities of life to individuals and families (Kela 2020). After that, income support and unemployment benefit variables were merged, while sickness allowance was merged with a disability pension variable.

#### Past-Year Gambling Behaviour

Gambling frequency was asked for 18 game types. The list included games provided by the Finnish gambling monopoly company, but also offshore games and games offered in Åland and in ferries between Finland, Sweden and Estonia were included. The overall gambling frequency was calculated based on the game type in which the gambler was the most active (Table 1). Gambling frequency, the number of game types gambled, and gambling mode were recoded into four categories.

Gambling severity was evaluated using the 14-item Problem and Pathological Gambling Measure (PPGM) (Williams & Volberg 2010). It was classified as: (1) problem gambling (incl. pathological gambling); (2) at-risk gambling; and (3) recreational gambling (incl. those gambling less than monthly). Cronbach’s alpha for PPGM was 0.842.

### Statistical Analysis

The data were weighted based on gender, age, education, and region of residence. Multinomial logistic regression analyses were used to estimate the association between the level of GE, socio-demographic factors, and gambling behaviour. The results are presented as odds ratios (ORs) and their 95 % confidence intervals (95 % CIs). The lowest GE group was used as the reference group. Multinomial logistic regression was conducted using IBM SPSS Statistic software version 27.0 (IBM Corp. Released 2020). The cumulative GE by PPGM and the cumulative GE by income tertiles were constructed using R software version 3.5.1 (R Core Team 2020). The respondents providing no GE information or PPGM information were excluded from the analyses.

## Results

**Fig. 1:**
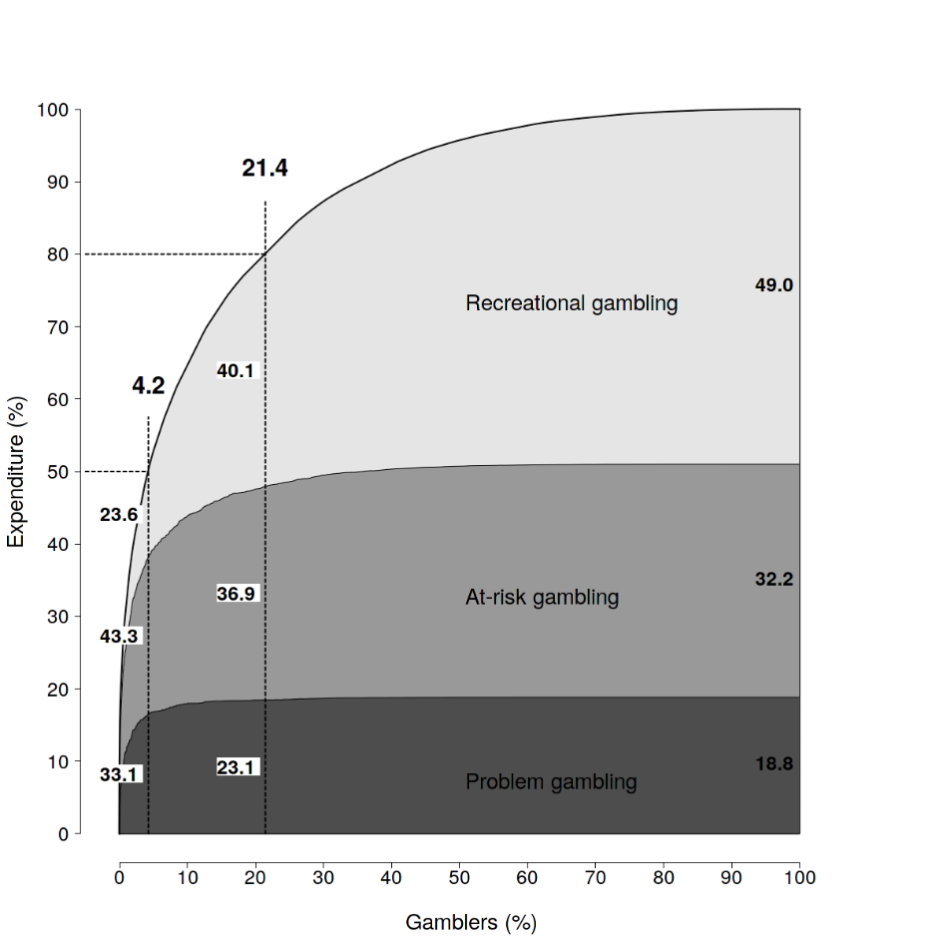
Cumulative GE by PPGM in 2016

Of the total GE, 18.8 % was produced by those with a gambling problem, 32.2 % by those with at-risk gambling pattern and 49.0 % by recreational gambling (Fig. 1). 4.2 % of gamblers produced 50.0 % of the total GE. Among these high-intensity consumers, 33.1 % of GE was produced by those with a gambling problem and 43.3 % by those with at-risk gambling pattern.

21.4 % of gamblers produced 80.0 % of the total GE. In this group, 23.1 % of GE was produced by those with a gambling problem, 36.9 % of GE by those with at-risk gambling, and 40.1 % by recreational gambling. The group with the lowest GE produced 20 % of the total GE.

### GE, Socio-Demographics and Gambling Behaviour

**Fig. 2:**
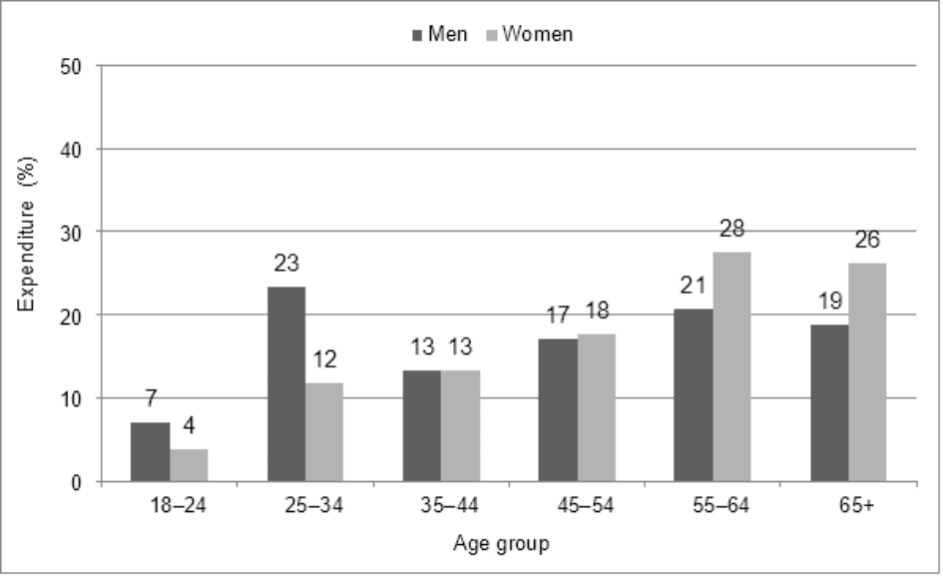
The total GE of men and women gamblers by age group in 2016

In the highest GE group (n = 255), the proportions of men and those aged 55 or older were higher compared with women and younger participants (Table 1). The intermediate GE group comprised of 20.3 % of gamblers (n = 1 179), and the lowest GE group of 75.5 % of gamblers (n = 4 204). In the lowest GE group, the proportion of 18–24-year-olds was the largest. GE differed between age groups (Fig. 2). Among men, 25–34-year-olds accounted for 23 % of men’s total GE, while the corresponding figure among women was only 12 %. On the other hand, 55-year-old or older women accounted for 54 % of women’s total GE, while the corresponding figure among men was 40 %.

**Table 1:** The gambling expenditure (GE) groups by socio-demographic factors and gambling behaviour in 2016, n (%)

	Highest expenditure		Intermediate expenditure		Lowest expenditure		
	n = 255 (4.2 %)		n = 1 179 (20.3 %)		n = 4 204 (75.5 %)		P-value
*Gender*							< 0.001
Men	203	(7.1)	779	(27.3)	1 867	(65.5)	
Women	52	(1.9)	399	(14.3)	2 338	(83.8)	
*Age*							< 0.001
65+	55	(4.8)	330	(28.3)	762	(66.4)	
55–64	68	(7.1)	268	(28.1)	617	(64.7)	
45–54	43	(4.2)	220	(21.5)	758	(74.2)	
35–44	38	(3.9)	161	(16.4)	780	(79.7)	
25–34	40	(4.1)	139	(14.4)	789	(81.5)	
18–24	10	(1.8)	60	(10.6)	498	(87.7)	
*Education level*							< 0.001
Missing	77	(6.6)	358	(30.7)	733	(62.8)	
Upper secondary	122	(5.2)	530	(22.5)	1 701	(72.3)	
Short-cycle tertiary education	23	(4.2)	111	(20.4)	411	(75.4)	
Bachelor or equivalent	17	(2.2)	105	(13.4)	662	(84.4)	
Master or doctoral degree or equivalent	15	(1.9)	74	(9.4)	698	(88.7)	
*Employment status*							< 0.001
Outside the labour force	13	(2.9)	55	(12.2)	382	(84.9)	
Retired based on age or service years	84	(5.9)	416	(29.5)	912	(64.6)	
Unemployed	28	(6.6)	91	(21.4)	307	(72.1)	
Employed	130	(3.9)	615	(18.4)	2 604	(77.8)	
*Net income tertiles*							0.001
T1 (lowest net income)	67	(3.9)	331	(19.3)	1 321	(76.8)	
T2	90	(4.6)	462	(23.8)	1 387	(71.5)	
T3 (highest net income)	98	(5.0)	385	(19.8)	1 466	(75.2)	
Missing	0	(0)	2	(6.1)	31	(93.9)	
*Income support or unemployment benefit*							0.223
Yes	51	(5.3)	186	(19.3)	725	(75.4)	
No	204	(4.4)	992	(21.2)	3 479	(74.4)	
*Disability pension or sickness allowance*							< 0.001
Yes	36	(9.5)	106	(28.0)	237	(62.5)	
No	219	(4.2)	1 073	(20.4)	3 968	(75.4)	
*Gambling frequency, past-year*							< 0.001
Daily	96	(37.6)	123	(48.2)	36	(14.1)	
Several times a week	93	(21.2)	229	(52.3)	116	(26.5)	
Once a week	55	(3.4)	633	(39.6)	911	(57.0)	
1–3 times a month or less	11	(0.3)	192	(5.8)	3 131	(93.9)	
*Game types gambled, past-year*							< 0.001
11–18 game types	46	(25.7)	64	(35.8)	69	(38.5)	
6–10 game types	117	(13.0)	335	(37.2)	449	(49.8)	
4–5 game types	41	(3.5)	310	(26.5)	818	(70.0)	
1–3 game types	50	(1.5)	465	(13.9)	2 830	(84.6)	
*Gambling mode, past-year*							< 0.001
Multi-mode	138	(8.4)	462	(28.2)	1 040	(63.4)	
Online only	38	(4.2)	222	(24.7)	639	(71.1)	
Land-based only	68	(2.4)	447	(15.5)	2 370	(82.1)	
Do not know / missing	12	(5.6)	47	(21.9)	156	(72.6)	
*Gambling severity, past-year*							< 0.001
Problem gambling	73	(45.9)	56	(35.2)	30	(18.9)	
At-risk gambling	91	(13.6)	277	(41.5)	299	(44.8)	
Recreational gambling	256	(4.6)	843	(17.6)	3 865	(80.5)	

The GE groups are categorised as follows: (1) spends EUR ≥ 40.0 per week and produces 50 % of the total GE (highest GE group), (2) spends EUR 10.0–39.9 per week and produces 30 % of the total GE (intermediate GE group), and (3) spends EUR ≤ 9.9 per week and produces 20 % of the total GE (lowest GE group)

Weighted based on gender, age, education, and region of residence. Statistical significance (p) was calculated using Pearson’s chi-squared test. The GE groups are: (1) spends EUR ≥ 40.0 per week and produces 50 % of the total GE (highest GE group), (2) spends EUR 10.0–39.9 per week and produces 30 % of the total GE (intermediate GE group), and (3) spends EUR ≤ 9.9 per week and produces 20 % of the total GE (lowest GE group)

In the highest GE group, the proportions of those with upper secondary education, the unemployed or those on income support, those on statutory retirement, those with the lowest net income, and those who had received disability pension or sickness allowance were higher than of the proportions of the other groups (Table 1). The proportions of those gambling several times a week and gambling six or more game types were higher. The participants who gambled multi-mode or online were more often in the highest GE group compared to those who gambled only land-based. In addition, the proportion of at-risk or problem gambling (ARPG) was higher than the proportion of recreational gamblers. The corresponding figures for those in the intermediate GE group were fairly similar.

### Multinomial Logistic Regression

Compared to the lowest GE group, those in the highest GE group had higher odds of being male, aged 25 or older, having upper secondary education level or information of their education was missing, having high income, and receiving disability pension or sickness allowance (Table 2). In addition, they had higher odds of gambling at least once a week, at least six game types or more, and having an ARPG pattern.

Compared to the lowest GE group, those in the intermediate GE group had higher odds of being male, aged 25 or older and having upper secondary education, short-cycle tertiary education, or information of their education was missing. Low income indicated lower odds of being in the intermediate GE group. Furthermore, gambling at least once a week, gambling at least four game types, gambling multi-mode and having and ARPG pattern had higher odds of being in the intermediate GE group.

**Table 2:** The association between gambling expenditure (GE) groups, socio-demographic factors and gambling behaviour in 2016: multinomial logistic regression

	Highest expenditure versus lowest expenditure			Intermediate expenditure versus lowest expenditure		
	OR	95 % CI	P-value	OR	95 % CI	P-value
*Gender*						
Men	*2.5*	*1.70, 3.73*	< 0.001	*1.8*	*1.55, 2.18*	< 0.001
Women	Ref.			Ref.		
*Age*						
65+	*40.9*	*12.20, 137.31*	< 0.001	*5.5*	*3.15, 9.73*	< 0.001
55–64	*27.5*	*9.94, 75.87*	< 0.001	*4.6*	*2.90, 7.32*	< 0.001
45–54	*9.1*	*3.32, 24.95*	< 0.001	*2.8*	*1.79, 3.01*	< 0.001
35–44	*5.3*	*1.94, 14.32*	0.001	*1.9*	*1.22, 3.05*	0.005
25–34	*7.5*	*2.81, 20.06*	< 0.001	*1.8*	*1.15, 2.81*	0.010
18–24	Ref.			Ref.		
*Education level*						
Missing	*2.5*	*1.17, 5.31*	0.018	*2.5*	*1.76, 3.54*	< 0.001
Upper secondary	*2.3*	*1.11, 4.58*	0.024	*2.3*	*1.66, 3.13*	< 0.001
Short-cycle tertiary education	1.5	0.64, 3.54	0.347	*1.5*	*1.04, 2.22*	0.031
Bachelor or equivalent	1.3	0.54, 3.14	0.555	1.4	0.98, 2.05	0.068
Master or Doctoral or equivalent	Ref.			Ref.		
*Employment status*						
Outside the labour force	1.6	0.64, 3.76	0.327	1.1	0.70, 1.66	0.730
Retired based on age or service years	1.1	0.53, 2.01	0.887	1.0	0.67, 1.41	0.922
Unemployed	1.4	0.67, 3.01	0.366	0.9	0.63, 1.39	0.749
Employed	Ref.			Ref.		
*Net income tertiles*						
T1 (low net income)	*0.6*	*0.39, 1.00*	0.049	*0.8*	*0.61, 0.96*	0.019
T2	Ref.			Ref.		
T3 (high net income)	*2.0*	*1.30, 3.15*	0.002	1.1	0.86, 1.32	0.575
*Income support or unemployment benefit*						
Yes	0.8	0.44, 1.40	0.788	0.8	0.63, 1.11	0.218
No	Ref.			Ref.		
*Disability pension or sickness allowance*						
Yes	*2.0*	*1.08, 3.83*	0.028	1.3	0.93, 1.86	0.128
No	Ref.			Ref.		
*Gambling frequency, past-year*						
Daily	*224.8*	*104.43, 483.67*	< 0.001	*24.9*	*16.36, 37.99*	< 0.001
Several times a week	*76.3*	*37.70, 155.00*	< 0.001	*15.2*	*11.38, 20.41*	< 0.001
Once a week	*10.0*	*5.05, 19.77*	< 0.001	*7.5*	*6.16, 9.02*	< 0.001
1–3 times a month or less	Ref.			Ref.		
*Game types gambled, past-year*						
11–18 game types	*18.5*	*9.01, 37.88*	< 0.001	*3.9*	*2.44, 6.27*	< 0.001
6–10 game types	*5.7*	*3.48, 9.23*	< 0.001	*3.1*	*2.41, 3.98*	< 0.001
4–5 game types	1.4	0.85, 2.35	0.182	*1.8*	*1.45, 2.21*	< 0.001
1–3 game types	Ref.			Ref.		
*Gambling mode, past-year*						
Multi-mode	1.4	0.92, 2.26	0.112	*1.4*	*1.08, 1.68*	0.007
Online	1.1	0.67, 1.86	0.692	1.2	0.98, 1.55	0.073
Land-based	Ref.			Ref.		
Do not know / missing	1.2	0.50, 2.88	0.685	1.2	0.77, 1.78	0.466
*Gambling severity, past-year*						
Problem gambling	*29.5*	*15.38, 56.64*	< 0.001	*3.7*	*2.16, 6.50*	< 0.001
At-risk gambling	*3.4*	*2.31, 5.07*	< 0.001	*1.8*	*1.42, 2.24*	< 0.001
Recreational gambling	Ref.			Ref.		
*R² (Nagelkerke)*		0.53				
*N*		5 564				

The GE groups are: (1) spends EUR ≥40.0 per week and produces 50% of the total GE (highest GE group), (2) spends EUR 10.0–39.9 per week and produces 30% of the total GE (intermediate GE group), and (3) spends EUR ≤9.9 per week and produces 20% of the total GE (lowest GE group). The lowest GE group is the reference group

Weighted based on gender, age, education, and region of residence. Note: OR = odds ratio, CI = confidence interval. The reference group is gamblers that spend EUR ≤ 9.9 per week and produce 20 % of the total GE. The GE groups are: (1) spends EUR ≥ 40.0 per week and produces 50 % of the total GE (highest GE group), (2) spends EUR 10.0–39.9 per week and produces 30 % of the total GE (intermediate GE group), and (3) spends EUR ≤ 9.9 per week and produces 20 % of the total GE (lowest GE group)

### Cumulative Weekly GE by Income Tertiles

**Fig. 3:**
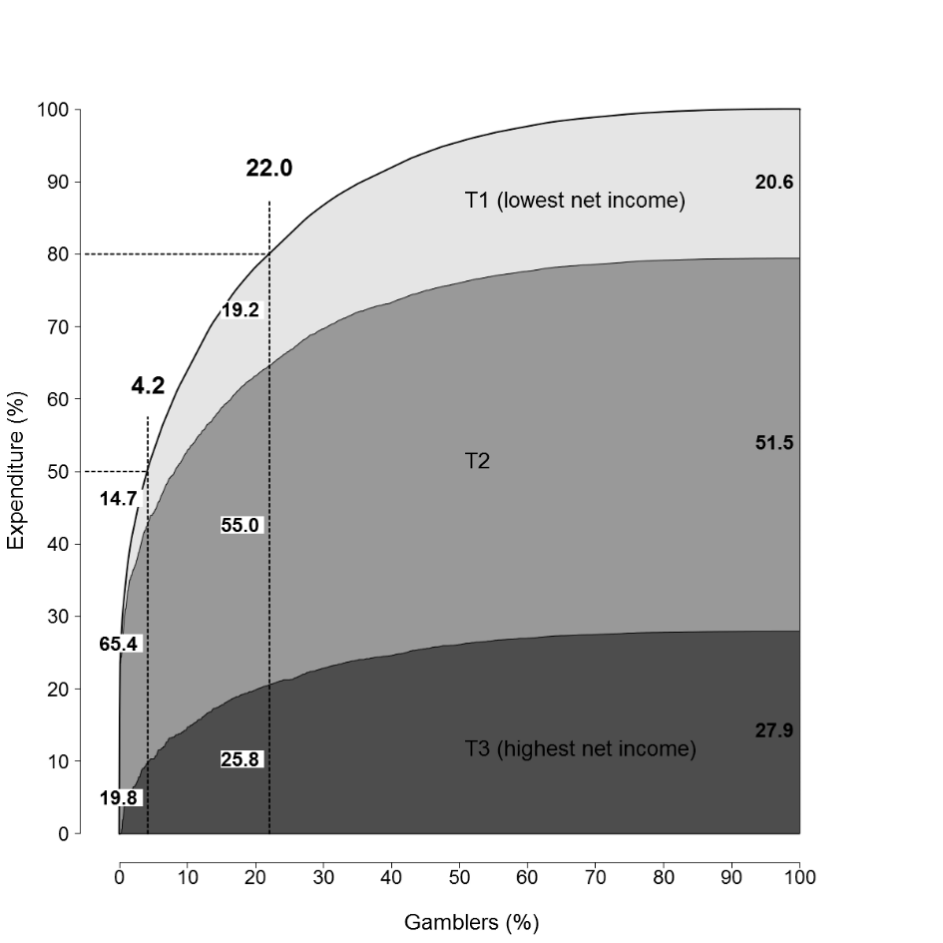
Cumulative expenditure by income tertiles in 2016

When cumulative weekly GE by income tertiles were examined, only past-year gamblers, those who had participated in both Waves and whose income information was available were included in the analysis (non-weighted). In 2016 (n = 2 140), 20.6 % of the total GE was produced by those with low net income (T1), 51.1 % of the total GE by those with intermediate net income (T2) and 27.9 % of the total GE by those with high net income (T3) (Fig. 3). In the group of 22.0 % that produced 80.0 % of the total GE, 19.2 % of GE was produced by those with low income, 55.0 % by those with intermediate income and 25.8 % of GE by those with high income. In the group of 4.2 % of gamblers that produced 50.0 % of the total GE, 14.7 % of GE was produced by those with low income, 65.4 % by those with intermediate income and 19.8 % by those with high income.

**Fig. 4:**
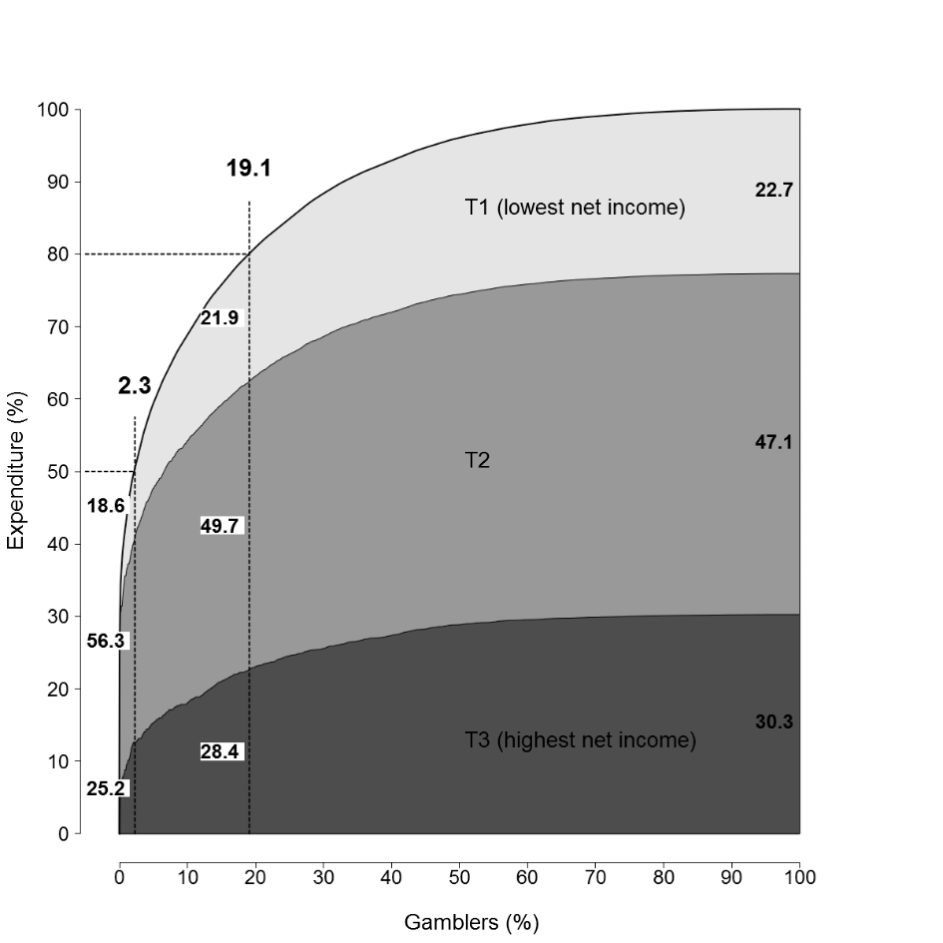
Cumulative expenditure by income tertiles in 2017

In 2017 (n = 2 090), 22.7 % of the total GE was produced by those with low income, 47.1 % by those with intermediate income and 30.3 % by those with high income (Fig. 4). In the group of 19.1 % that produced 80.0 % of the total GE, 21.9 % of GE was produced by those with low income, 49.7 % of GE by those with intermediate income and 28.4 % of GE by those with high income. In the group of 2.3 % that produced 50.0 % of the total GE, 18.6 % of GE was produced by those in with low income, 56.3 % of GE by those with intermediate income and 25.2 % of GE by those with high income.

## Discussion

In the present study, a small group of gamblers (4.2 %) produced half (50.0 %) of the total GE in 2016. This result is in line with earlier research indicating that GE is highly concentrated (Salonen et al., 2017; Salonen et al., 2019; Salonen et al., 2020; Williams et al., 2011). In Finland, corresponding figures have varied from 2.2 to 5.2 % of gamblers that produce half of the gambling revenue (Salonen et al., 2017; Salonen et al., 2019; Salonen et al., 2020).

In this study, of the total GE, half of the money came from persons with ARPG. Among the small group of high-intensity consumers, 76.4 % of the revenue came from ARPG. Gamblers with disordered gambling typically spend more money on gambling than they intended to, are unable to control their gambling, and take debts in order to continue gambling. In addition, they typically continue gambling to recover previous losses, often with increasing the size of bets (American Psychiatric Association 2013). Despite the fact that higher GE was clearly linked with ARPG, all GE groups had encountered ARPG. Among people with a low socio-economic status, even small losses can cause GRH.

Overall, respondents in the low GE group differed from those in the intermediate and high GE groups. Those in the highest or intermediate GE group were more likely to be male which is in accordance with earlier studies (McCormack et al., 2014; Roukka & Salonen 2020; Salonen et al., 2020). This may be related to the nature of the games men favour. While men tend to prefer strategic games such as poker and sport betting, particularly online (Wardle et al., 2011), women tend to prefer non-strategic games such as scratch cards (Salonen & Raisamo 2015). These strategic game types have fast gambling speed and high stakes, which expose men to higher GE than women.

Compared to the lowest GE group, those in the highest or intermediate GE group were less likely to be 18–24-year-old. Older adults (≥ 55 years) spent the most. Older adults still in employment may have more money to spend on gambling (Castrén et al., 2018). Among the retired adults, fixed incomes and limited prospects of future earnings make them a vulnerable group (Subramaniam et al., 2014). This is especially the case among older women, as they often face a higher poverty risk than older men (Nygård et al., 2017). In the present study, 55 years or older women accounted for 54 % of women’s total GE, while the corresponding figure among men was 40 %. Opposite results have also been observed in terms of the association between age and GE. Previous studies have found that GE increases until the middle age and starts to drop after that (Mikesell 1991; Scott & Garen 1994).

Those in the highest or intermediate GE group had a lower education level than those in the lowest GE group. This finding is in line with prior research suggesting that low education is associated with higher GE (Worthington et al., 2003; Davidson et al., 2016; Salonen et al., 2017; Salonen et al., 2018a). According to a Finnish study conducted in 2017, 70.9 % of the total GE was produced by those with upper secondary or short-cycle tertiary education. Those with a bachelor’s degree or higher produced 13.0 % of total GE. (Salonen et al., 2017.) An Australian study found that the difference of gambling spending between educational groups is particularly high in EGMs. Those with low education lost on average 6.5 times more on EGMs than those with a degree. (Davidson et al., 2016.) The density of EGMs is the highest in areas of socio-economic disadvantage (Wardle et al., 2014; Raisamo et al., 2019). Overall, EGM density and high GE are positively correlated (Vasiliadis et al., 2013), which highlights the need for further research taking into account the game types.

Those in the highest GE group had a higher net income than those in the lowest GE group. This is consistent with previous results (Williams et al., 2011; Salonen et al., 2020). However, studies have also shown that those with lower income spend more on gambling (Beckert & Lutter 2009; Bol et al., 2014). In relation to the gambler’s net income, people with lower income have been found to spent more on gambling than those with higher net income (Castrén et al., 2018). Employment status and income support or unemployment benefit were not associated with the level of GE. Those who had received disability pension or sickness allowance had a higher GE than of those without these benefits. This result is supported by previous studies (Salonen et al., 2019; Roukka & Salonen 2020). Overall, poor health is shown to be associated with higher GE (Salonen et al., 2017).

Compared to the lowest GE group, those in the highest or intermediate GE group gamble more often, gamble more game types and more often engage in multi-mode gambling. These findings are consistent with what has been found in earlier studies (Salonen & Raisamo 2015; Salonen et al., 2017; Salonen et al., 2020). Gambling frequency has been shown to be the strongest indicator of high GE (Salonen et al., 2018a).

The proportion of intermediate income was higher in the group of gamblers that produced 50.0 % of the total GE than in the group of all gamblers that produced 100.0 % of the GE. It is noteworthy that the proportion of the first net income tertile is not considerably lower in the small group of gamblers that produced 50.0 % of the total GE than in the group of all gamblers that produced 100.0 % of the GE. This result was observed for both 2016 and 2017.

### Strengths and Limitations

Self-reported data were used as in most gambling studies (Shaffer et al., 2010). Studies evaluating the accuracy of self-reported data with actual data provided by gambling operator indicate that respondents tend to underestimate the amount spent on gambling (Braverman et al., 2014; Auer & Griffiths 2017), but self-reported loss still correlates with the actual loss (Auer & Griffiths 2017). Furthermore, the respondents did not consistently indicate a favourable distortion of their gambling losses or gains, as they underestimated or overestimated their gambling outcomes (Braverman et al., 2014). The format of the question being asked and how the respondents are instructed can affect the responses (Blaszczynski et al., 2006; Wood & Williams 2007). Herein, the participants were asked to estimate their GE based on the frequency of their choice, which may have contributed into rather small amount of missing data (see Salonen & Raisamo 2015; Salonen et al., 2020). Finally, those with higher gambling losses and those experiencing GRHs have more difficulties estimating their spending on gambling accurately (Braverman et al., 2014; Auer & Griffiths 2017). Gambling severity was not based on medical diagnosis but was evaluated by using Problem and Pathological Gambling Measure (PPGM).

## Conclusions

This study confirmed that GE is highly concentrated on the small group of gamblers. In the group of high-intensity consumers, the most of the GE was produced by those with ARPG patterns. In addition to the highest GE group, ARPG occurred in the lowest and intermediate GE group. It is noteworthy that those with ARPG often face other challenges as well, such as financial difficulties, substance abuse and mental health problems (Hodgins et al., 2011; Lorains et al., 2011; Castrén et al., 2013). These challenges are further aggravated by gambling. Participants in the lowest GE group differed from those in the intermediate and highest GE group in terms of their socio-demographic background and gambling behaviour. Cumulative weekly GE by income tertiles remained fairly stable between the two study years. In order to make gambling policy more responsible, the group of high-intensity consumers should be considered better in strategies to prevent and reduce the gambling-related financial, health-related, and social harm.

## Electronic Supplementary Material

Below is the link to the electronic supplementary material.

Supplementary Material 1

## Data Availability

The Gambling Harms Survey 2016 and 2017 datasets are available from the Finnish Social Science Data Archive (https://www.fsd.tuni.fi/en/).
