# Supplementary material for: Socio-Demographic Factors, Gambling Behaviour, and the Level of Gambling Expenditure: A Population-Based Study
Source: J Gambl Stud. 2021 Oct 4;38(4):1093–109. doi: 10.1007/s10899-021-10075-6 (PMC9653360; doi:10.1007/s10899-021-10075-6)
Supplement: Supplementary file 1 — Supplementary Material 1 [file 10899_2021_10075_MOESM1_ESM.docx]

**Abstract**

The aim of this study was to examine the relationship between socio-demographic factors, gambling behaviour, and the level of gambling expenditure. The data were drawn from the population-based Gambling Harms Survey 2016 and 2017 conducted in Finland. The data was combined with register-based variables. Past-year gamblers were included (Wave 1; n = 5 805, both Waves; n = 2 165). The study showed that of the 4.2% of gamblers that produced 50.0% of the total GE in 2016, 33.1% of the GE was produced by those with a gambling problem and 43.3% by those with at-risk gambling pattern. Compared to gamblers in the lowest GE group, those in the highest GE group were more likely to be men, aged 25 or older, with upper secondary education, have a high income, be on disability pension or sickness allowance, be frequent gamblers, gambling at least six game types, and showing at-risk and problem gambling patterns. Cumulative weekly GE by income tertiles remained fairly stable between the years. The results suggest that GE is highly concentrated. Among the small group of high-intensity consumers, the majority of the revenue comes from at-risk and problem gambling. Participants in the low GE group differ from those in the intermediate and high GE groups in terms of socio-demographics and gambling behaviour.

**INTRODUCTION**

Gambling environment has changed rapidly. Gambling is increasingly available and more advertised, and new gambling products are constantly being developed. People also spend more money on gambling than before (Statista 2020). Finland’s per capita gambling expenditure (GE) rate is one of the highest in Europe (Economist 2017). Spending for gambling is highly concentrated (Salonen, Kontto, Alho & Castrén 2017; Salonen, Lind, Castrén, Lahdenkari, Kontto, Selin, Hellman & Järvinen-Tassopoulos 2019; Salonen, Hagfors, Lind & Kontto 2020; Williams, Belanger & Arthur 2011). This raises a question of how much of the gambling revenue is produced by individuals from different socio-demographic backgrounds and, especially, by those with a gambling problem.

Overall, men tend to spend more money on gambling than women (McCormack, Shorter & Griffiths 2014; Davidson et al. 2016; Castrén et al. 2018). This has been observed for both younger and older adults (Merkouris, Thomas, Shandley, Rodda, Oldenhof & Dowling 2016; Molinaro, Benedetti, Scalese, Bastaini, Fortunato, Cerrai, Canale, Chomynova, Elekes, Feijao, Fotiou, Kokkevi, Kraus, Rupsiene, Monshouwer, Nociar, Strizek & Lazar 2018). GE increases with age (Tan, Yen, Nayga & Rodolfo 1991), although some studies suggest that GE increases only until the middle age and starts to drop after that (Mikesell 1991; Scott & Garen 1994).

Moreover, low socio-economic status is associated with high GE (Davidson, Rodgers, Markham & Taylor-Rodgers 2016; Salonen, Kontto, Perhoniemi, Alho & Castrén 2018a). To date, a limited number of studies have investigated the relationship between GE and receipt of social security benefits (Worthington 2001; MacDonald, McMullan & Perrier 2004). A Canadian survey showed that households with income support were less likely to gamble. With the exception of one jurisdiction, households that received income support spend a lower proportion of their income on gambling. (MacDonald, McMullan & Perrier 2004.) However, gambling problems are more common among those who receive income support or who are unemployed (McMillen, Marshall, Ahmed & Wenzel 2003). Moreover, gambling opportunities are concentrated in the socio-economically disadvantaged areas (Wardle, Keily, Astbury & Reith 2014; Raisamo, Toikka, Selin & Heiskanen 2019).

Studies conducted in different countries have shown that although high income groups spend more on gambling, lower income groups contribute proportionally more (Beckert & Lutter 2009; Canale, Vieno & Griffiths 2016; Castrén, Kontto, Alho & Salonen 2018; Roukka & Salonen 2019). In general, among those with low income, a large fraction of their income spent on gambling often leads to financial harm (Welte, Barnes, Wieczorek, Tidwell & Parker 2004; Freund & Morris 2006), since they have fewer financial resources to cover up their losses. This may lead to debt taking (Sulkunen, Babor, Örnberg, Egerer, Hellman, Livingstone, Marionneau & Nikkinen 2019). A Finnish population survey showed that among help-seeking gamblers, 42% had a debt problem or a debt spiral (Salonen, Hellman, Latvala & Castrén 2018b). Gambling-related harms (GRH) include financial consequences, such as over-indebtedness or losing funds intended for household expenses. In some cases, gamblers may lose all their assets, even their homes. (Sulkunen et al. 2019.) In addition, gambling may cause work-related problems and job loss; housing instability or homelessness; and criminal behaviour (Williams, Rehm & Stevens 2011; Gattis & Cunninham-Williams 2011; Lind, Kääriäinen & Kuoppamäki 2015; Eby, Mitchell, Gray, Provolt, Lorys, Fortune & Goodie 2016).

High gambling frequency is associated with high GE (LaPlante, Nelson, LaBrie & Shaffer 2009). The number of game types gambled affects GE, as those who gamble six or more game types spend the most. Furthermore, online gambling is associated with higher GE than land-based gambling (Salonen et al. 2017). Weekly gambling on horse races, online poker, EGMs and offshore games are linked with higher GE (Salonen et al. 2018b). EGMs cause more GRHs than other game types (Peluuri 2016; Binde, Romild & Volberg 2017).

High GE does not directly mean that a gambler experience GRH, but it is associated with GRH (Currie, Hodgins, Wang, El-Guebaly, Wynne & Chen 2006; Langham, Thorne, Browne, Donaldson, Rose & Rockloff 2016). A relatively large proportion of the gambling revenue comes from those with a gambling problem (e.g., Grinols & Omorov 1996; Lesieur 1998; Volberg, Moore, Christiansen, Cummings & Banks 1998; Volberg, Gerstein, Christiansen & Baldridge 2011; Williams & Wood 2004; Wood & Williams 2007; Salonen et al. 2017; Fiedler, Kairouz, Costes & Weissmüller 2019). However, in previous studies, this proportion have ranged widely from 14% (Volberg, Gerstein, Christiansen & Baldridge 2011) to 52% (Grinols & Omorov 1996). In addition to several methodological differences, the proportion of gambling revenue derived from those with a gambling problem depends on the game type, with lower proportions for lotteries and higher proportions for electronic gaming machines (EGM) (Volberg et al. 1998; Wood & Williams 2007).

Until 2016, the Finnish gambling policy was based on a three-party monopoly system. These operators were merged into a single state-owned company in 2017. Under the Lotteries Act, the country’s gambling system’s main objective is to prevent and reduce the gambling-related financial, health and social harm (Finlex 2011). Most of the Finnish gambling company’s profits are channelled through the state or non-governmental organisations to promote the public good. The use of gambling revenues for good purposes is often used as a justification for the existence of this monopoly, in which all citizens are seen as beneficiaries.

As discussed above, previous studies have investigated the association between gambling expenditure, socio-demographic factors, and gambling behaviour. Nevertheless, very little is known about the small group of gamblers that produces the most of the gambling revenue. This study aims to investigate the relationship between socio-demographic factors (education, employment status, net income, and social security benefits), gambling behaviour (gambling frequency, number of game types gambled, gambling mode, and gambling severity), and the level of GE. The gamblers with the highest GE are compared to other gamblers.

**METHODS**

**Design and participants**

The data were drawn from the Gambling Harms Survey 2016–2017 conducted in Finland [4]. Finland is the only European Union country that still maintains a gambling monopoly; this makes Finland an interesting case. In addition, these data are longitudinal, which allows us to analyse individual-level changes. At Wave 1, a total of 20,000 permanent residents of selected areas were randomly selected from the national Population Information System. The inclusion criteria were: 1) age 18 or over; 2) Finnish or Swedish as first language; and 3) living in Uusimaa, Pirkanmaa or Kymenlaakso. At Wave 1, a total of 4 611 participants gave their permission to be re-contacted.

**Data collection**

The study was conducted by the Finnish Institute for Health and Welfare in collaboration with the University of Helsinki. The data were collected by Statistics Finland between 9 January and 26 March 2017 (Wave 1) and between 15 January and 30 April 2018 (Wave 2) by using online and postal survey. The focus was on gambling in 2016 and 2017.

At Wave 1, a participant information sheet was sent to the potential participants. The first reminder was sent by mail. To 25–44-years-olds, the reminder was also sent by text message. The third and fourth reminder included a postal survey. At Wave 2, the postal survey was sent to participants that had responded via postal survey at Wave 1. Similarly, an invitation with two reminders was sent to those participants that had answered to online survey at Wave 1. All invitations included a link to online survey.

At Wave 1, a total of 7 186 persons participated in the survey (response rate 36%). At Wave 2, there were 2 624 participants, and the response rate was 57% of eligible participants, who responded to Wave 1 survey and gave a permission for re-contact. At Wave 1, the response rate was lower in men than in women (Salonen et al. 2017); whereas at Wave 2 men responded more actively than women (Salonen et al. 2019). Overall, older and highly educated persons participated most actively.

Past-year gamblers were included (Wave 1; n = 5 805, both Waves; n = 2 165). Using national ID numbers, the survey data were linked to the Statistics Finland’s register data on socio-demographic factors.

**Measures**

*Gambling expenditure*

GE was measured as follows: ‘Think about the year 2016 (or 2017). Estimate the amount of money that you spent on gambling on average per week, per month or during the year (in Euros).’ All GE figures were transformed into weekly GE. To categorise three groups, the cumulative distribution of total GE was created by sorting GE in descending order. Then, the cumulative distribution was explored to find the point where half of the total GE was cumulated. Lastly, based on the Pareto Principle (Juran 1975), which assumes that for many phenomena roughly 80% of consequences are due to 20% of the causes, the cumulative distribution was explored to find the point where 80% of total GE was cumulated. Gamblers were divided into three groups as follows: 1) produces 50% of the total GE and spend EUR ≥40.0 per week (i.e., the highest GE group), 2) produces 30% of the total GE and spend EUR 10.0–39.9 per week (i.e., the intermediate GE group), and 3) produces 20% of the total GE and spend EUR ≤9.9 per week (i.e., the lowest GE group).

The highest GE at Wave 2 was considered as an outlier and replaced as follows: firstly, all gamblers in both Waves with increased GE were selected from the dataset. Then, the average growth of GE between the waves was calculated for them. The outlier was replaced by multiplying the respondents’ GE in Wave 1 by the average growth of GE.

*Socio-demographic factors*

Socio-demographic factors were derived from Statistics Finland’s registers and recoded (Table 1). They included gender, age, educational level, employment status, and personal net income tertiles. Furthermore, the amount of income support, unemployment benefit and sickness allowance were dichotomised (yes/no). In Finland, income support is a last-resort form of financial aid, which covers some of the basic necessities of life to individuals and families (Kela 2020). After that, income support and unemployment benefit variables were merged, while sickness allowance was merged with a disability pension variable.

*Past-year gambling behaviour*

Gambling frequency was asked for 18 game types. The list included games provided by the Finnish gambling monopoly company, but also offshore games and games offered in Åland and in ferries between Finland, Sweden and Estonia were included. The overall gambling frequency was calculated based on the game type in which the gambler was the most active (Table 1). Gambling frequency, the number of game types gambled, and gambling mode were recoded into four categories.

Gambling severity was evaluated using the 14-item Problem and Pathological Gambling Measure (PPGM) (Williams & Volberg 2010). It was classified as: 1) problem gambling (incl. pathological gambling); 2) at-risk gambling; and 3) recreational gambling (incl. those gambling less than monthly). Cronbach’s alpha for PPGM was 0.842.

**Statistical analysis**

The data were weighted based on gender, age, education, and region of residence. Multinomial logistic regression analyses were used to estimate the association between the level of GE, socio-demographic factors, and gambling behaviour. The results are presented as odds ratios (ORs) and their 95% confidence intervals (95% CIs). The lowest GE group was used as the reference group. Multinomial logistic regression was conducted using IBM SPSS Statistic software version 27.0 (IBM Corp. Released 2020). The cumulative GE by PPGM and the cumulative GE by income tertiles were **constructed** using R software version 3.5.1 (R Core Team 2020). The respondents providing no GE information or PPGM information were excluded from the analyses.

**RESULTS**

Of the total GE, 18.8% was produced by those with a gambling problem, 32.2% by those with at-risk gambling pattern and 49.0% by recreational gambling (Figure 1). 4.2% of gamblers produced 50.0% of the total GE. Among these high-intensity consumers, 33.1% of GE was produced by those with a gambling problem and 43.3% by those with at-risk gambling pattern.

[Fig. 1 Cumulative GE by PPGM in 2016]

21.4% of gamblers produced 80.0% of the total GE. In this group, 23.1% of GE was produced by those with a gambling problem, 36.9% of GE by those with at-risk gambling, and 40.1% by recreational gambling. The group with the lowest GE produced 20% of the total GE.

*GE, socio-demographics and gambling behaviour*

In the highest GE group (n = 255), the proportions of men and those aged 55 or older were higher compared with women and younger participants (Table 1). The intermediate GE group comprised of 20.3% of gamblers (n = 1 179), and the lowest GE group of 75.5% of gamblers (n = 4 205). In the lowest GE group, the proportion of 18–24-year-olds was the largest. GE differed between age groups (Figure 2). Among men, 25–34-year-olds accounted for 23% of men’s total GE, while the corresponding figure among women was only 12%. On the other hand, 55-year-old or older women accounted for 54% of women’s total GE, while the corresponding figure among men was 40%.

[Table 1 The GE groups by socio-demographic factors and gambling behaviour in 2016]

[Fig. 2 The total GE of men and women gamblers by age group in 2016]

In the highest GE group, the proportions of those with upper secondary education, the unemployed or those on income support, those on statutory retirement, those with the lowest net income, and those who had received disability pension or sickness allowance were higher than of the proportions of the other groups (Table 1). The proportions of those gambling several times a week and gambling six or more game types were higher. The participants who gambled multi-mode or online were more often in the highest GE group compared to those who gambled only land-based. In addition, the proportion of at-risk or problem gambling (ARPG) was higher than the proportion of recreational gamblers. The corresponding figures for those in the intermediate GE group were fairly similar.

*Multinomial logistic regression*

Compared to the lowest GE group, those in the highest GE group had higher odds of being male, aged 25 or older, having upper secondary education level or information of their education was missing, having high income, and receiving disability pension or sickness allowance (Table 2). In addition, they had higher odds of gambling at least once a week, at least six game types or more, and having an ARPG pattern.

Compared to the lowest GE group, those in the intermediate GE group had higher odds of being male, aged 25 or older and having upper secondary education, short-cycle tertiary education, or information of their education was missing. Low income indicated lower odds of being in the intermediate GE group. Furthermore, gambling at least once a week, gambling at least four game types, gambling multi-mode and having and ARPG pattern had higher odds of being in the intermediate GE group.

[Table 2 The association between the GE groups, socio-demographic factors and gambling behaviour in 2016: multinomial logistic regression]

**Cumulative weekly GE by income tertiles**

When cumulative weekly GE by income tertiles were examined, only past-year gamblers, those who had participated in both Waves and whose income information was available were included in the analysis (non-weighted). In 2016 (n = 2 140), 20.6% of the total GE was produced by those with low net income (T1), 51.1% of the total GE by those with intermediate net income (T2) and 27.9% of the total GE by those with high net income (T3) (Figure 3). In the group of 22.0% that produced 80.0% of the total GE, 19.2% of GE was produced by those with low income, 55.0% by those with intermediate income and 25.8% of GE by those with high income. In the group of 4.2% of gamblers that produced 50.0% of the total GE, 14.7% of GE was produced by those with low income, 65.4% by those with intermediate income and 19.8% by those with high income.

[Fig. 3 Cumulative expenditure by income tertiles in 2016]

In 2017 (n = 2 090), 22.7% of the total GE was produced by those with low income, 47.1% by those with intermediate income and 30.3% by those with high income (Figure 4). In the group of 19.1% that produced 80.0% of the total GE, 21.9% of GE was produced by those with low income, 49.7% of GE by those with intermediate income and 28.4% of GE by those with high income. In the group of 2.3% that produced 50.0% of the total GE, 18.6% of GE was produced by those in with low income, 56.3% of GE by those with intermediate income and 25.2% of GE by those with high income.

[Fig. 4 Cumulative expenditure by income tertiles in 2017]

**DISCUSSION**

In the present study, a small group of gamblers (4.2%) produced half (50.0%) of the total GE in 2016. This result is in line with earlier research indicating that GE is highly concentrated (Salonen et al. 2017; Salonen et al. 2019; Salonen et al. 2020; Williams, Belanger & Arthur 2011). In Finland, corresponding figures have varied from 2.2% to 5.2% of gamblers that produce half of the gambling revenue (Salonen et al. 2017; Salonen et al. 2019; Salonen et al. 2020).

In this study, of the total GE, half of the money came from persons with ARPG. Among the small group of high-intensity consumers, 76.4% of the revenue came from ARPG. Gamblers with disordered gambling typically spend more money on gambling than they intended to, are unable to control their gambling, and take debts in order to continue gambling. In addition, they typically continue gambling to recover previous losses, often with increasing the size of bets (American Psychiatric Association 2013). Despite the fact that higher GE was clearly linked with ARPG, all GE groups had encountered ARPG. Among people with a low socio-economic status, even small losses can cause GRH.

Overall, respondents in the low GE group differed from those in the intermediate and high GE groups. Those in the highest or intermediate GE group were more likely to be male which is in accordance with earlier studies (McCormack et al. 2014; Roukka & Salonen 2020; Salonen, Lind, Hagfors & Castrén 2020). This may be related to the nature of the games men favour. While men tend to prefer strategic games such as poker and sport betting, particularly online [54], women tend to prefer non-strategic games such as EGMs, bingo, and lottery (Salonen & Raisamo 2015). These strategic game types have fast gambling speed and high stakes, which expose men to higher GE than women.

Compared to the lowest GE group, those in the highest or intermediate GE group were less likely to be 18–24-year-old. Older adults (≥55 years) spent the most. Older adults still in employment may have more money to spend on gambling (Castrén et al. 2018). Among the retired adults, fixed incomes and limited prospects of future earnings make them a vulnerable group (Subramaniam, Wang, Soh, Vaingankar, Chong, Browning & Thomas 2014). This is especially the case among older women, as they often face a higher poverty risk than older men (Nygård, Härtull, Wentjärvi & Jungerstam 2017). In the present study, 55 years or older women accounted for 54% of women’s total GE, while the corresponding figure among men was 40%. Opposite results have also been observed in terms of the association between age and GE. Previous studies have found that GE increases until the middle age and starts to drop after that (Mikesell 1991; Scott & Garen 1994).

Those in the highest or intermediate GE group had a lower education level than those in the lowest GE group. This finding is in line with prior research suggesting that low education is associated with higher GE (Worthington, Brown, Crawford & Pickernell 2003; Davidson et al. 2016; Salonen et al. 2017; Salonen et al. 2018a). According to a Finnish study conducted in 2017, 70.9% of the total GE was produced by those with upper secondary or short-cycle tertiary education. Those with a bachelor’s degree or higher produced 13.0% of total GE. (Salonen et al. 2017.) An Australian study found that the difference of gambling spending between educational groups is particularly high in EGMs. Those with low education lost on average 6.5 times more on EGMs than those with a degree. (Davidson et al. 2016.) The density of EGMs is the highest in areas of socio-economic disadvantage (Wardle et al. 2014; Raisamo et al. 2019). Overall, EGM density and high GE are positively correlated (Vasiliadis, Jackson, Christensen & Francis 2013), which highlights the need for further research taking into account the game types.

Those in the highest GE group had a higher net income than those in the lowest GE group. This is consistent with previous results (Williams et al. 2011; Salonen et al. 2020). However, studies have also shown that those with lower income spend more on gambling (Beckert & Lutter 2009; Bol, Lancee & Steijn 2014). In relation to the gambler’s net income, people with lower income have been found to spent more on gambling than those with higher net income (Castrén et al. 2018). Employment status and income support or unemployment benefit were not associated with the level of GE. Those who had received disability pension or sickness allowance had a higher GE than of those without these benefits. This result is supported by previous studies (Salonen et al. 2019; Roukka & Salonen 2020). Overall, poor health is shown to be associated with higher GE (Salonen et al. 2017).

Compared to the lowest GE group, those in the highest or intermediate GE group gamble more often, gamble more game types and more often engage in multi-mode gambling. These findings are consistent with what has been found in earlier studies (Salonen & Raisamo 2015; Salonen et al. 2017; Salonen et al. 2020). Gambling frequency has been shown to be the strongest indicator of high GE (Salonen et al. 2018a).

The proportion of intermediate income was higher in the group of gamblers that produced 50.0% of the total GE than in the group of all gamblers that produced 100.0% of the GE. It is noteworthy that the proportion of the first net income tertile is not considerably lower in the small group of gamblers that produced 50.0% of the total GE than in the group of all gamblers that produced 100.0% of the GE. This result was observed for both 2016 and 2017.

*Strengths and limitations*

Self-reported data were used as in most gambling studies (Shaffer, Allyson, LaPlante, Nelson & Labrie 2010). Studies evaluating the accuracy of self-reported data with actual data provided by gambling operator indicate that respondents tend to underestimate the amount spent on gambling (Braverman, Tom & Shaffer 2014; Auer & Griffiths 2017), but self-reported loss still correlates with the actual loss (Auer & Griffiths 2017). Furthermore, the respondents did not consistently indicate a favourable distortion of their gambling losses or gains, as they underestimated or overestimated their gambling outcomes (Braverman, Tom & Shaffer 2014). The format of the question being asked and how the respondents are instructed can affect the responses (Blaszczynski, Ladouceur, Goulet & Savard 2006; Wood & Williams 2007). Herein, the participants were asked to estimate their GE based on the frequency of their choice, which may have contributed into rather small amount of missing data (see Salonen & Raisamo 2015; Salonen et al. 2020). Finally, those with higher gambling losses and those experiencing GRHs have more difficulties estimating their spending on gambling accurately (Braverman et al. 2014; Auer & Griffiths 2017). Gambling severity was not based on medical diagnosis but was evaluated by using Problem and Pathological Gambling Measure (PPGM).

*Conclusions*

This study confirmed that GE is highly concentrated on the small group of gamblers. In the group of high-intensity consumers, the most of the GE was produced by those with ARPG patterns. In addition to the highest GE group, ARPG occurred in the lowest and intermediate GE group. It is noteworthy that those with ARPG often face other challenges as well, such as financial difficulties, substance abuse and mental health problems (Hodgins, Stea & Grant 2011; Lorains, Colishaw & Thomans 2011; Castrén, Basnet, Salonen, Pankakoski, Ronkainen, Alho & Lahti 2013). These challenges are further aggravated by gambling. Participants in the lowest GE group differed from those in the intermediate and highest GE group in terms of their socio-demographic background and gambling behaviour. Cumulative weekly GE by income tertiles remained fairly stable between the two study years. In order to make gambling policy more responsible, the group of high-intensity consumers should be considered better in strategies to prevent and reduce the gambling-related financial, health-related, and social harm.

**References**

American Psychiatric Association. (2013). Diagnostic and Statistical Manual of Mental Disorders: DSM-5, 5th edn. Arlington, VA: American Psychiatric Association.

Auer, M. & Griffiths, M. (2017). Self-reported losses versus actual losses in online gambling: An empirical study. *Journal of Gambling Studies* *33*(3):795–806. https://doi.org/10.1007/s10899-016-9648-0

Beckert, J. & Lutter, M. (2009). The Inequality of fair play: Lottery Gambling and Social Stratification in Germany. *European Sociological Review* *25*(4):475–488. https://doi.org/10.1093/esr/jcn063

Binde, P., Romild, U. & Volberg, R. (2017). Forms of gambling, gambling involvement and problem gambling: evidence from a Swedish population survey. *International Journal of Gambling Studies* *17*(3):490–507. https://doi.org/10.1080/14459795.2017.1360928

Blaszczynski, A., Ladouceur, R., Goulet, A. & Savard, C. (2006). How much do you spend on gambling?: Ambiguities in questionnaire items assessing expenditure. *International Journal of Gambling Studies* *6*(2):123–128. https://doi.org/10.1080/14459790600927738

Bol, T., Lancee, B. & Steijn, S. (2014). Income inequality and gambling: A panel study in the United States (1980–1997). *Sociological Spectrum* *34*(1):61–75. https://doi.org/10.1080/02732173.2014.857196

Braverman, J., Tom, M. & Shaffer, H. (2014). Accuracy of self-reported versus actual online gambling wins and losses. *Psychological Assessment* *26*(3):865–877. https://doi.apa.org/doi/10.1037/a0036428

Canale, N., Vieno, A. & Griffiths, M. (2016). The extent and distribution of gambling-related harms and the prevention paradox in a British population survey. *Journal of Behavioural Addiction* *5*(2):204–212. https://doi.org/10.1556/2006.5.2016.023

Castrén, S., Basnet, S., Salonen, A.H., Pankakoski, M., Ronkainen, J.E., Alho, H. & Lahti, T. (2013). Factors associated with disordeded gambling in Finland. *Substance Abuse Treatment, Preventation, and Policy* *24*(8). https://doi.org/10.1186/1747-597X-8-24

Castrén, S., Kontto, J., Alho, H. & Salonen, A.H. (2018). The relationship between gambling expenditure, socio-demographics, health-related correlates and gambling behaviour – a cross-sectional population survey in Finland. *Addiction 113*(1):91–106. https://doi.org/10.1111/add.13929

Currie, S., Hodgins, D., Wang, J., El-Guebaly, N., Wynne, H. & Chen, S. (2006). Risk of harm among gamblers in the general population as a function of level of participation in gambling activities. *Addiction* *101*(4):570–580. https://doi.org/10.1111/j.1360-0443.2006.01392.x

Davidson, T., Rodgers, B., Markham, F. & Taylor-Rodgers, E. (2016). Gambling expenditure in the ACT (2014): By level of problem gambling, type of activity, and socioeconomic and demographic characteristics. Final report. Available at: https://www.gamblingandracing.act.gov.au/__data/assets/pdf_file/0010/982774/2014-Gambling-Expenditure.pdf. Accessed 25 May 2021. http://dx.doi.org/10.11575/PRISM/9512

Eby, L.T., Mitchell, M.E., Gray, C.J., Provolt, L., Lorys, A., Fortune, E. & Goodie, A.S. (2016). Gambling-related Problems Across Life Domains: An Exploratory Study of Non-Treatment-Seeking Weekly Gamblers. *Community, Work & Family* *19*(5):604–620. https://doi.org/10.1080/13668803.2015.1112255

Economist. (2017). The world’s biggest gamblers. Daily Chart. Graphic detail. Charts, maps and infographics. Available at: https://www.economist.com/graphic-detail/2017/02/09/the-worlds-biggest-gamblers. Accessed 27 September 2019.

Fiedler, I., Kairouz, S., Costes, J.M. & Weissmüller, K. (2019). Gambling spending and its concentration on problem gamblers. *Journal of Business Research* *98*(20):82–91. https://doi.org/10.1016/j.jbusres.2019.01.040

Finlex. (2011). Lotteries Act 14 §20.5.2011/575.

Freund, E. & Morris, I. (2006). Gambling and Income Inequality in the States. *Policy Studies Journal* *34*(2):265–276. https://doi.org/10.1111/j.1541-0072.2006.00169.x

Grinols, E.L. & Omorov, J.D. (1996). Who loses when casino wins? *Illionois Business Review* *53*:7–11.

Gattis, M. & Cunninham-Williams, R. (2011). Housing Stability and Problem Gambling: Is There a Relationship? *Journal of Social Service Research* *37*(5):490–499. https://doi.org/10.1080/01488376.2011.598716

Hodgins, D., Stea, J. & Grant, J. (2011). Gambling disorders. *The Lancet* *378*(9806):1874–1884. https://doi.org/10.1016/S0140-6736(10)62185-X

IBM Corp. Released 2020. IBM SPSS Statistics for Windows, Version 27.0. Armonk, NY: IBM Corp.

Juran, J. (1975). The non-Pareto principle; mea culpa. *Quality Progress* *8*(5):8­–9.

Kela. (2020). Overview of social assistance. Available at: https://www.kela.fi/web/en/social-assistance-overviewhttps://www.kela.fi/web/en/social-assistance-overview. Accessed 21 December 2020.

Langham, E., Thorne, H., Browne, M., Donaldson, P., Rose, J. & Rockloff, M. (2016). Understanding gambling related harm: a proposed definition, conceptual framework and taxonomy of harms. *BMC Public Health* *16*(80). https://doi.org/10.1186/s12889-016-2747-0

LaPlante, D., Nelson, S., LaBrie, R. & Shaffer, H. (2009). The relationships between disordered gambling, type of gambling, and gambling involvement in the British gambling prevalence survey 2007. *European Journal of Public Health* *21*(4):532–537. https://doi.org/10.1093/eurpub/ckp177

Lesieur, H.R. (1998). Costs and treatment of pathological gambling. *The ANNALS of the American Academy of Political and Social Science* *556*:153–171. https://doi.org/10.1177%2F0002716298556001012

Lind, K., Kääriäinen, J. & Kuoppamäki, S. (2015). From problem gambling to crime. Findings from the Finnish National Police Information System. *Journal of Gambling Issues 30*:98–123. https://doi.org/10.4309/jgi.2015.30.10

Lorains, F.K., Colishaw, S. & Thomans, S.A. (2011). Prevalence of comorbid disorders in problem and pathological gambling: systematic review and meta-analysis of population surveys. *Addiction* *106*(3):490–498. https://doi.org/10.1111/j.1360-0443.2010.03300.x

MacDonald, M., McMullan, J. & Perrier, D. (2004). Gambling households in Canada. *Journal of Gambling Studies* *20*(3):187–236. https://doi.org/10.1023/B:JOGS.0000040277.85569.a1

McCormack, A., Shorter, G.W. & Griffiths, M.D. (2014). An Empirical Study of Gender Differences in Online Gambling*. Journal of Gambling Studies* *30*(1),71–88. https://doi.org/10.1007/s10899-012-9341-x

McMillen, J., Marshall, D., Ahmed, E. & Wenzel, M. (2004). 2003 Victorian longitudinal community attitudes survey. Gambling Research Panel (GRP). Report No. 6. Available at: https://openresearch-repository.anu.edu.au/bitstream/1885/45189/3/VicLongCommAS_FinalComplete_03.pdf. Accessed 25 May 2021.

Merkouris, S., Thomas, A., Shandley, K., Rodda, S., Oldenhof, E. & Dowling, N. (2016). An update on gender differences in the characteristics associated with problem gambling: A systematic review. *Current Addiction Reports* *3*(3):254–267. https://doi.org/10.1007/s40429-016-0106-y

Mikesell, J. (1991). Lottery expenditure in a non-lottery state. *Journal of Gambling Studies* *7*(2):89–98. https://doi.org/10.1023/B:JOGS.0000040277.85569.a1

Molinaro, S., Benedetti, E., Scalese, M, Bastaini, L, Fortunato, L., Cerrai, S., Canale, N., Chomynova, P., Elekes, Z., Feijao, F., Fotiou, A., Kokkevi, A., Kraus, L., Rupsiene, L., Monshouwer, K., Nociar, A., Strizek, J. & Lazar, T. (2018). Prevalence of youth gambling and potential influence of substance use and other risk factors across 33 European countries: First results from the 2015 ESPAD study. *Addiction* *113*(10):1862–1873. https://doi.org/10.1111/add.14275

Nygård, M., Härtull, C., Wentjärvi, A. & Jungerstam, S. (2017). Poverty and Old Age in Scandinavia: A Problem of Gendered Injustice? Evidence from the 2010 GERDA Survey in Finland and Sweden. *Social Indicators Research* *132*(2):681–698. https://doi.org/10.1007/s11205-016-1313-6

Peluuri. (2016). Peluurin vuosiraportti 2015–2016. [Peluuri’s Annual Report 2015–2016]. Available at: https://www.peluuri.fi/peluuri/peluurin-vuosiraportit. Accessed 27 April 2021.

Raisamo, S., Toikka, A., Selin, J. & Heiskanen, M. (2019). The density of electronic gambling machines and area-level socioeconomic status in Finland: a country with a legal monopoly on gambling and a decentralised system of EGMs. *BMC Public Health* *19*(1). https://doi.org/10.1186/s12889-019-7535-1

R Core Team. (2020). R: A language and environment for statistical computing. R Foundation for Statistical Computing, Vienna, Austria. URL https://www.R-project.org/.

Roukka, T. & Salonen, A.H. (2020). The Winners and the Losers: Tax Incidence of Gambling in Finland. *Journal of Gambling Studies 36*(4):1183–1204. https://doi.org/10.1007/s10899-019-09899-0

Salonen, A.H., Hagfors, H., Lind, K. & Kontto, J. (2020). Rahapelaaminen ja peliongelmat –Suomalaisten rahapelaaminen 2019. [Gambling and problem gambling – Finnish Gambling 2019]. National Institute for Health and Welfare (THL). Report 8/2020. Helsinki 2020.

Salonen, A.H., Hellman, M., Latvala, T. & Castrén, S. (2018b). Gambling participation, gambling habits, gambling-related harm, and opinions on gambling advertising in Finland in 2016. *Nordic Studies on Alcohol and Drugs 35*(3):215–234. https://doi.org/10.1177%2F1455072518765875

Salonen, A.H., Kontto, J., Alho, H. & Castrén, S. (2017). Suomalaisten rahapelikulutus – keneltä rahapeliyhtiöiden tuotot tulevat? [Gambling expenditure in Finland – who contributes the most to the profits of the gambling industry?]. *Yhteiskuntapolitiikka 82*(5):549–559.

Salonen, A.H, Kontto, J., Perhoniemi, R., Alho, A. & Castrén, S. (2018a). Gambling expenditure by game type among weekly gamblers in Finland. *BMC Public Health* *697*(18). https://doi.org/10.1186/s12889-018-5613-4

Salonen, A.H., Lind, K., Castrén, S., Lahdenkari, M., Kontto, J., Selin, J., Hellman, M. & Järvinen-Tassopoulos, J. (2019). Rahapelaaminen, rahapelihaitat ja rahapelien markkinointiin liittyvät mielipiteet kolmessa maakunnassa. [Gambling Harms Survey 2016–2017: Gambling, gambling-related harm and opinions on gambling marketing in three regions in connection with the reform of the Finnish gambling monopoly]. National Institute for Health and Welfare (THL). Report 4/2019. Helsinki 2019.

Salonen, A.H., Lind, K., Hagfors, H., Castrén, S. & Kontto, J. (2020). Rahapelaaminen, peliongelmat ja rahapelaamiseen liittyvät asenteet ja mielipiteet vuosina 2007–2019. [Gambling, problem gambling and attitudes and opinions towards gambling in 2007–2019]. National Institute for Health and Welfare (THL). Report 18/2020. Helsinki 2020.

Salonen, A.H. & Raisamo, S. (2015). Suomalaisten rahapelaaminen 2015. Rahapelaaminen, rahapeliongelmat ja rahapelaamiseen liittyvät asenteet ja mielipiteet 15–74-vuotiailla. [Finnish gambling 2015. Gambling, gambling problems, and attitudes and opinions on gambling among Finns aged 15–74]. National Institute for Health and Welfare (THL). Report 16/2015. Helsinki 2015.

Scott, F. & Garen, J. (1994). Probability of purchase, amount of purchase, and the demographic incidence of the lottery tax. *Journal of Public Economics* *54*:121–143. https://doi.org/10.1016/0047-2727(94)90073-6

Shaffer, H., Allyson, J., LaPlante, D., Nelson, S. & Labrie, R. (2010). Toward a paradigm shift in Internet gambling research: From opinion and self-report to actual behavior. *Addiction Research Theory 18*(3):270–283. https://doi.org/10.3109/16066350902777974

Statista. (2020). Global gambling market gross gaming yield (GGY) from 2001 to 2019 [internet]. Available at: https://www.statista.com/statistics/253416/global-gambling-market-gross-win/. Accessed 21 on October 2020.

Subramaniam, M., Wang, P., Soh, P., Vaingankar, J.A., Chong, S.A., Browning, C.J. & Thomas, S.A. (2014). Prevalence and determinants of gambling disorder among older adults: a systematic review. *Addictive Behaviours* *41*:199–209. https://doi.org/10.1016/j.addbeh.2014.10.007

Sulkunen, P., Babor, T., Örnberg, J., Egerer, M., Hellman, M., Livingstone, C., Marionneau, V., Nikkinen, J., Orford, J., Room, R. & Rossow, I. (2019). Setting Limits: Gambling, Science and Public Policy. Oxford University Press. https://doi.org/10.1093/oso/9780198817321.001.0001

Tan, A.K.G., Yen, S.T., Nayga, R.M. Jr. & Rodolfo, M. (2010). Socio-demographic determinants of gambling participation and expenditures: evidence from Malaysia. *International Journal of Consumer Studies* *34*(3):316–325. https://psycnet.apa.org/doi/10.1111/j.1470-6431.2009.00856.x

Vasiliadis, S., Jackson, A., Christensen, D. & Francis, K. (2013). Physical accessibility of gaming opportunity and its relationship to gaming involvement and problem gambling: A systematic review. *Journal of Gambling Issues* *28, 1­–46*. https://psycnet.apa.org/doi/10.4309/jgi.2013.28.2

Volberg, R., Gerstein, D.R., Christiansen, E. & Baldridge, J. (2011). Assessing self-reported expenditures on gambling. *Managerial and Decision Economics* *22*:77–96. https://doi.org/10.1002/mde.999

Volberg, R., Moore, W., Christiansen, E., Cummings, W. & Banks, S. (1998). Unaffordable losses: Estimating the proportion of gambling revenues derived from problem gamblers. *Gaming Law Review 2*(4):349–360. https://doi.org/10.1089/glr.1998.2.349

Wardle, H., Keily, R., Astbury, G. & Reith, G. (2014). Risky Places? Mapping gambling machine density and socio-economic deprivation. *Journal of Gambling Studies* *30*(1):201–212. https://doi.org/10.1007/s10899-012-9349-2

Wardle, H., Moody, A., Spence, S., Orford, J., Griffiths, M., Volberg, R., Jotangia, D., Griffiths, M., Hussey, D. & Dobbie, F. (2011). British gambling prevalence survey 2010. National Centre for Social Research. The Stationery Office; London.

Welte, J., Barnes, G., Wieczorek, W., Tidwell, M.C. & Parker, J. (2004). Risk Factors for Pathological Gambling. *Addictive Behaviours* *29*(2):323–335. https://doi.org/10.1016/j.addbeh.2003.08.007

Williams, R. J., Belanger, Y. D. & Arthur, J. N. (2011). Gambling in Alberta: History, Current Status and Socioeconomic Impacts. Final report to the Alberta Gambling Research Institute. Edmonton, Alberta 2011. Available at: https://prism.ucalgary.ca/bitstream/handle/1880/48495/SEIGA%20FINAL%20REPORT-Apr2.pdf?sequence=3&isAllowed=y. Accessed 4 April 2021.

Williams, R. J., Rehm, J. & Stevens, R.M.G. (2011). The Social and Economic Impacts of Gambling. Final Report to the Canadian Interprovincial Consortium for Gambling Research. Available at: https://opus.uleth.ca/bitstream/handle/10133/1286/SEIG_FINAL_REPORT_2011.pdf?sequence=1&isAllowed=y. Accessed 25 May 2021.

Williams, R.J. & Volberg, R.A. (2010). Best practices in the population assessment of problem gambling. Guelph: Ontario Problem Gambling Research Centre. 2010.

Williams, R. & Wood, R. (2004). The Proportion of Gaming Revenue Derived from Problem Gamblers: Examining the Issues in a Canadian Context. *Analyses of Social Issues and Public Policy* *4*(1):33–45. https://psycnet.apa.org/doi/10.1111/j.1530-2415.2004.00033.x

Wood, R. & Williams, R.J. (2007). How much money do you spend on gambling? The comparative validity of question wordings used to assess gambling expenditure. *International Journal of Social Research Methodology* *10*(1):63–77. https://doi.org/10.1080/13645570701211209

Worthington, A.C. (2001). Implicit finance in gambling expenditures: Australian evidence on socio-economic and demographic tax incidence. *Public Finance Review* *29*(4):326–342. https://doi.org/10.1177%2F109114210102900403

Worthington, A.C., Brown, K., Crawford, M. & Pickernell, D. (2003). Socioeconomic and demographic determinants of household gambling in Australia. Discussion paper no. 156. School of Economics and Finance. Brisbane, Qld: Queensland University of Technology. Available at: http://eprints.qut.edu.au/329/1/Discussion_Paper_Worthington,_Brown,_Crawford,_Pickernell_-_No_156.pdf. Accessed 15 January 2021.
